# Genetics of osteoarthritis

**DOI:** 10.1016/j.joca.2021.03.002

**Published:** 2022-05

**Authors:** G. Aubourg, S.J. Rice, P. Bruce-Wootton, J. Loughlin

**Affiliations:** Biosciences Institute, Newcastle University, Newcastle Upon Tyne, UK

**Keywords:** Genetics, Epigenetics, SNPs, GWAS, DNA methylation, Functional analysis

## Abstract

Osteoarthritis genetics has been transformed in the past decade through the application of large-scale genome-wide association scans. So far, over 100 polymorphic DNA variants have been associated with this common and complex disease. These genetic risk variants account for over 20% of osteoarthritis heritability and the vast majority map to non-protein coding regions of the genome where they are presumed to act by regulating the expression of target genes. Statistical fine mapping, *in silico* analyses of genomics data, and laboratory-based functional studies have enabled the identification of some of these targets, which encode proteins with diverse roles, including extracellular signaling molecules, intracellular enzymes, transcription factors, and cytoskeletal proteins. A large number of the risk variants correlate with epigenetic factors, in particular cartilage DNA methylation changes *in cis*, implying that epigenetics may be a conduit through which genetic effects on gene expression are mediated. Some of the variants also appear to have been selected as humans adapted to bipedalism, suggesting that a proportion of osteoarthritis genetic susceptibility results from antagonistic pleiotropy, with risk variants having a positive role in joint formation but a negative role in the long-term health of the joint. Although data from an osteoarthritis genetic study has not yet directly led to a novel treatment, some of the osteoarthritis associated genes code for proteins that have available therapeutics. Genetic investigations are therefore revealing fascinating fundamental insights into osteoarthritis and can expose options for translational intervention.

## Introduction

Comprehensive molecular genetic investigations into multifactorial human diseases have been a scientific highlight of the past decade. Such analyses have garnered insights into fundamental aspects of disease pathophysiology, identifying gene targets and biological pathways for therapeutic intervention. These genetic studies involve the genome-wide association scan (GWAS) of DNA variants, principally mapping alleles at single nucleotide polymorphisms (SNPs), in cases and controls. The use of large cohorts, often involving hundreds of thousands of individuals, and high-density SNP genotyping arrays supplemented by statistical imputation has enabled tens of thousands of DNA variants to be associated with a broad range of diseases^1^.

Osteoarthritis (OA) has been subjected to such detailed genetic investigation and each year new OA susceptibility risk loci are reported, along with subsequent mechanistic investigations that help to clarify how genetic risk impacts the cells and tissues of the articulating joint. In this review we summarize the current status of OA genetics, from discovery of risk loci and their functional investigation, through to epigenetics and the clinical utility of the new knowledge.

### OA is a polygenic disease

Investigators conducting OA GWAS have focused on cases diagnosed with the typical age-related form of the disease, in which onset occurs from the fifth decade of life. Furthermore, individuals with post-traumatic OA, or another obviously non-genetic cause of disease, are often excluded from analyses. The logic here is that the case group will then consist of individuals who are more likely to have a genetic component underlying their disease. This is the most common form of OA, which consequently confers the highest burden upon society. Selection of OA cases has involved a range of diagnostic tools including X-ray evidence of joint space narrowing, joint pain, or the need to replace the diseased joint due to severe end-stage OA. Cases have been sub-categorized based on disease at particular skeletal sites (principally knees, hips, and hands) and GWAS have been performed both by joint site stratification, and by combining all cases. Studies have encompassed European cohorts, individuals of European descent, Asian cohorts, and African Americans. To increase confidence in the results, studies are collaborative and usually involve a discovery component followed by replication and meta-analysis. Such rigor has enabled geneticists to identify 124 SNPs associated with OA to date (Table I)^2^, ^3^, ^4^, ^5^, ^6^, ^7^, ^8^, ^9^, ^10^, ^11^, ^12^, ^13^, ^14^, ^15^, ^16^, ^17^, ^18^, ^19^, ^20^, ^21^, ^22^, ^23^, ^24^, ^25^. These SNPs encompass 95 independent loci spread across the genome, with some loci (for example, at the type XI collagen gene *COL11A1*) having several SNPs marking separate associations at the locus. OA is therefore a highly polygenic disease.

**Table I:** The 124 SNPs significantly associated with osteoarthritis risk. The nearest protein coding gene to the SNP is named. EA, effect allele; NEA, non-effect allele; OR, odds ratio; ns, not stated. The physical location of the SNP (hg19 genome assembly) is reported along with the chromatin-state description of the region in the Roadmap ChIP-seq dataset in mesenchymal stem cells (MSCs, E006), cultured chondrocytes (E046), adipose-derived MSCs (E025), cultured adipocytes (E023), and osteoblasts (E129)

Chromosome	Association SNP	Nearerst protein coding gene	EA/NEA	OR	Variant (chromatin state)	Position (hg19)	*P*-value	Causal gene	Reference
1	rs3753841	*COL11A1*	A/G	1.08	Missense (Transcribed)	10,33,79,918	5.20 × 10^−10^		^22^
	rs2126643	*COL11A1*	C/T	1.10	Intronic (Transcribed)	10,34,04,384	2.10 × 10^−14^		^22^
	rs4338381	*COL11A1*	A/G	1.10	Intronic (Promoter)	10,35,72,927	4.37 × 10^−15^		^24^
	chr1:150214028	*ANP32E*	C/CT	1.03	Intergenic	15,02,14,028	2.54 × 10^−8^		^24^
	rs550034492	*RABGAP1L*	TA(17)/T	1.03	Intronic (Transcribed)	17,41,92,403	1.05 × 10^−8^		^24^
	rs11583641	*COLGALT2*	C/T	1.08	3′UTR (Transcribed)	18,39,06,245	5.58 × 10^−10^	*COLGALT2*	^24^
	rs2820436	*ZC3H11B*	A/C	1.07	Intergenic (Enhancer)	21,96,40,680	2.01 × 10^−9^		^21^
	rs2785988	*SLC30A10*	A/C	1.08	Intergenic (Repressed)	21,97,44,138	3.90 × 10^−10^		^22^
	rs2820443	*SLC30A10*	C/T	1.06	Intergenic (Enhancer)	21,97,53,509	2.01 × 10^−9^		^21^
				1.06			6.01 × 10^−11^		^24^
	rs10916199	*ZNF678*	A/G	ns	Intronic (Heterochromatin)	22,79,02,472	2.4 × 10^−13^	*WNT9A*	^25^
	rs10218792	*KIF26B*	G/T	1.04	Intronic (Transcribed)	24,57,50,932	2.03 × 10^−8^		^24^
2	rs2061027	*LTBP1*	A/G	1.04	Intronic (Transcribed)	33,43,336	3.16 × 10^−13^		^24^
	rs2061026	*LTBP1*	A/G	1.06	Intronic (Enhancer)	33,43,549	1.40 × 10^−11^		^22^
	rs2862851	*TGFA*	C/T	ns	Intronic (Repressed)	7,07,12,802	5.20 × 10^−11^		^14^
	rs3771501	*TGFA*	*A/G*	1.06	Intronic (Repressed)	7,07,17,653	1.66 × 10^−8^		^21^
				1.05			4.24 × 10^−16^		^24^
	rs12470967	*SDPR*	A/G	1.06	Intergenic	19,26,71,981	1.50 × 10^−8^		^24^
	rs62182810	*RAPH1*	A/G	1.03	Intronic (Transcribed)	20,43,87,482	1.65 × 10^−9^		^24^
3	rs7639618	*COL6A4P1*	G/A	1.43	Missense (Repressed)	1,52,16,429	7.3 × 10^−11^		^3^
	rs62262139	*RBM6*	A/G	1.04	Intronic (Transcribed)	5,00,22,049	9.09 × 10^−11^		^24^
	rs11177	*GNL3*	A/G	1.12	Missense (Transcribed)	5,27,21,305	1.25 × 10^−10^		^8^
	rs6976	*GNL3*	T/C	1.12	3′UTR (Transcribed)	5,27,28,804	7.24 × 10^−11^		^8^
	rs3774355	*ITIH1*	A/G	1.09	Intronic (Repressed)	5,28,17,778	8.20 × 10^−14^		^24^
	rs678	*ITIH1*	T/A	1.08	Missense (Repressed)	5,28,20,981	1.60 × 10^−9^		^22^
	rs12107036	*TP63*	G/A	1.21	Intronic	18,96,00,160	2.15 × 10^−3^		^8^
4	rs11732213	*SLBP*	T/C	1.06	Intronic (Transcribed)	17,04,244	8.81 × 10^−10^		^24^
	rs1913707	*RAB28*	A/G	1.08	Intergenic	1,30,39,440	2.96 × 10^−11^		^24^
	rs34811474	*ANAPC4*	G/A	1.04	Intronic (Transcribed)	2,54,08,838	2.17 × 10^−9^		^24^
	rs11335718	*ANXA3*	-/C	1.11	Intronic (Transcribed)	7,95,28,543	4.26 × 10^−8^		^21^
	rs13107325	*SLC39A8*	T/C	1.10	Missense (Transcribed)	10,31,88,709	8.29 × 10^−19^		^24^
5	rs10471753	*PIK3R1*	G/C	ns	Intergenic (Enhancer)	6,78,18,952	3.80 × 10^−9^		^14^
	rs35611929	*AP3B1*	A/G	1.06	Intronic (Transcribed)	7,74,67,824	8.29 × 10^−19^		^24^
	rs3884606	*FGF18*	G/A	1.04	Intronic (Enhancer)	17,08,71,074	8.25 × 10^−9^		^24^
6	rs1800562	*HFE*	G/A	1.95	Missense (Transcribed)	2,60,93,141	5.0 × 10^−14^		^22^
	rs115740542	*HIST1H2BC*	C/T	1.06	Intergenic (Promoter)	2,61,23,502	8.59 × 10^−9^		^24^
	rs10947262	*BTNL2*	C/T	1.31	Intronic (Repressed)	3,23,73,312	5.0 × 10^−9^		^4^
	rs7775228	*HLA-DQB1*	T/C	1.34	Intergenic (Repressed)	3,26,58,079	2.43 × 10^−8^		^4^
	rs9277552	*HLA-DPB1*	C/T	1.06	3′UTR (Transcribed)	3,30,55,501	2.37 × 10^−10^		^24^
	rs12154055	*CDC5L*	G/A	1.03	Intergenic	4,44,49,697	2.71 × 10^−8^		^24^
	rs10948155	*SUPT3H/RUNX2*	C/T	ns	Intergenic	4,46,87,987	5.20 × 10^−11^	*RUNX2*	^14^
	rs10948172	*SUPT3H/RUNX2*	G/A	1.14	Intronic (Enhancer)	4,47,77,961	7.92 × 10^−8^	*RUNX2*	^8^
							9.00 × 10^−11^		^22^
	rs2396502	*SUPT3H/RUNX2*	C/A	1.09	Intronic (Transcribed)	4,53,57,699	2.12 × 10^−12^		^24^
	rs1997995	*SUPT3H/RUNX2*	G/A	1.09	Intronic (Transcribed)	4,53,74,183	1.1 × 10^−11^		^22^
	rs12206662	*SUPT3H/RUNX2*	G/A	ns	Intronic (Transcribed)	4,53,76,221	1.3 × 10^−9^		^14^
	rs80287694	*BMP5*	G/A	1.12	Intronic	5,56,36,940	2.66 × 10^−9^		^24^
	rs12209223	*FILIP1*	A/C	1.16	Intronic	7,61,64,589	2.9 × 10^−15^		^22^
				1.17			3.88 × 10^−16^		^24^
	rs9350591	*FILIP1*	T/C	1.18	Intergenic	7,62,41,527	2.42 × 10^−9^		^8^
7	rs143083812	*SMO*	T/C	2.84	Missense (Transcribed)	1,28,84,410	7.90 × 10^−12^		^22^
	rs11764536	*HDAC9*	C/A	1.26	Intronic	1,84,09,993	1.60 × 10^−9^		^22^
	rs788748	*IGFBP3*	A/G	0.70	Intergenic	4,60,26,181	2.0 × 10^−8^		^13^
	rs11409738	*DYNC1L1*	TA/T	1.04	Intronic (Transcribed)	9,57,19,834	2.13 × 10^−10^		^24^
	rs3815148	*COG5*	C/A	1.14	Intronic (Transcribed)	10,69,38,420	4.11 × 10^−9^		^5^
	rs4730250	*DUS4L*	G/A	1.17	Intronic (Transcribed)	10,72,07,695	9.20 × 10^−9^		^11^
	rs7792864	*RNF32*	C/G	2.35	Intergenic	15,63,46,087	4.00 × 10^−9^		^17^
8	rs330050	*PPP1R3B*	G/C	1.04	Intergenic (Repressed)	90,87,679	1.93 × 10^−11^		^24^
	rs4733724	*GSDMC*	A/G	1.11	Intergenic (Enhancer)	13,01,23,728	7.20 × 10^−12^		^22^
	rs60890741	*GSDMC*	C/CA	1.11	Intronic	13,07,68,503	4.50 × 10^−9^		^24^
	rs11780978	*PLEC*	A/G	1.13	Intronic (Transcribed)	14,50,34,852	1.98 × 10^−9^	*PLEC*	^21^
9	rs10116772	*GLIS3*	C/A	1.03	Intronic (Transcribed)	42,90,541	3.71 × 10^−8^		23
	rs10974438	*GLIS3*	A/C	1.03	Intronic (Transcribed)	42,91,928	1.34 × 10^−8^		^24^
	rs116882138	*MOB3B*	A/G	1.25	Intergenic	2,73,13,557	5.09 × 10^−8^		^21^
	rs1078301	*COL27A1*	T/A	1.07	Intergenic (Repressed)	11,69,09,146	1.4 × 10^−10^		^22^
	rs919642	*COL27A1*	T/A	1.05	Intergenic (Repressed)	11,69,11,147	8.55 × 10^−15^		^24^
	rs1330349	*TNC*	C/G	1.08	Intronic (Transcribed)	11,78,40,742	4.10 × 10^−11^		^24^
	rs2480930	*TNC*	A/G	1.09	Intronic (Transcribed)	11,78,42,307	6.60 × 10^−12^		^22^
	rs4836732	*ASTN2*	C/T	1.20	Intronic (Transcribed)	11,92,66,695	6.11 × 10^−10^		^8^
	rs13283416	*ASTN2*	G/T	1.10	Intronic	11,93,01,607	5.3 × 10^−14^		^22^
	rs34687269	*ASTN2*	A/T	1.09	Intronic	11,94,84,132	1.67 × 10^−12^		^24^
	rs10760442	*LMX1B*	G/A	1.09	Intronic (Repressed)	12,93,83,900	7.60 × 10^−12^		^22^
	rs62578127	*LMX1B*	C/T	1.09	Intronic (Repressed)	12,93,86,860	2.77 × 10^−12^		^24^
11	rs17659798	*METTL15*	A/C	1.06	Intergenic	2,88,74,997	2.06 × 10^−10^		^24^
	rs11031191	*DCDC5*	T/G	1.03	Intergenic	3,07,74,280	1.42 × 10^−8^		^24^
	rs2070852	*F2*	C/G	ns	Intronic (Transcribed)	4,67,44,925	4.7 × 10^−8^		^25^
	rs10896015	*LTBP3*	G/A	1.09	Intronic (Enhancer)	6,53,23,725	7.70 × 10^−10^		^22^
				1.08			2.74 × 10^−9^		^24^
	rs34419890	*SPTBN2*	T/C	1.13	Intergenic (Repressed)	6,65,01,624	1.99 × 10^−8^		^24^
	rs1149620	*TSKU*	T/A	1.04	Intronic (Transcribed)	7,65,06,572	6.93 × 10^−10^		^24^
12	rs4764133	*ERP27*	T/C	ns	Intergenic	1,50,64,363	1.80 × 10^−15^	*MGP*	^18^
				ns			4.8 × 10^−14^		^25^
	rs10492367	*PTHLH/KLHL42*	T/G	1.14	Intergenic	2,80,14,970	1.25 × 10^−24^		^24^
	rs10843013	*PTHLH/KLHL42*	C/A	1.14	Intergenic	2,80,25,196	5.4 × 10^−19^		^22^
	rs12049916	*CCDC91*	G/A	ns	Intronic	2,83,59,985	2.00 × 10^−8^		^25^
	rs79056043	*LRIG3*	G/A	1.18	Intronic (Transcribed)	5,82,89,598	1.33 × 10^−9^		^24^
	rs317630	*CPSF6*	T/C	1.04	Intronic (Transcribed)	6,96,37,847	1.97 × 10^−8^		^24^
	rs11105466	*ATP2B1*	A/G	1.04	Intergenic (Enhancer)	9,03,26,919	2.16 × 10^−8^		^24^
	rs2171126	*CRADD*	T/C	1.03	Intronic (Transcribed)	9,41,67,220	9.07 × 10^−10^		^24^
	rs835487	*CHST11*	G/A	1.13	Intronic	10,50,60,767	1.64 × 10^−^^8^		^8^
	rs11059094	*MLXIP*	T/C	1.08	Intronic (Transcribed)	12,26,06,837	7.38 × 10^−11^		^24^
	rs1060105	*SBNO1*	C/T	1.07	Missense (Transcribed)	12,38,06,219	1.9 × 10^−8^		^22^
	rs56116847	*SBNO1*	A/G	1.06	Intronic (Transcribed)	12,38,35,233	3.19 × 10^−10^		^24^
	rs4765540	*FAM101A*	C/T	1.08	3′UTR (Transcribed)	12,47,99,642	3.4 × 10^−9^		^22^
13	rs11842874	*MCF2L*	A/G	1.17	Intergenic (Repressed)	11,36,94,509	2.04 × 10^−8^		^7^
15	rs35912128	*USP8*	T/-	1.08	Intronic (Transcribed)	5,07,31,132	2.18 × 10^−8^		^24^
	rs4775006	*ALDH1A2*	A/C	1.06	Intergenic (Enhancer)	5,82,15,727	8.40 × 10^−10^		^24^
	rs3204689	*ALDH1A2*	C/G	1.46	3′UTR (Transcribed)	5,82,46,802	3.99 × 10^−10^	*ALDH1A2*	^12^
	rs12901372	*SMAD3*	C/G	1.08	Intronic (Enhancer)	6,70,78,168	5.60 × 10^−11^		^22^
	rs12901071	*SMAD3*	A/G	1.08	Intronic (Enhancer)	6,73,70,389	3.12 × 10^−10^		^20^
	rs35206230	*CSK*	T/C	1.04	Intergenic (Transcribed)	7,50,97,780	1.48 × 10^−12^		^24^
16	rs9930333	*FTO*	G/T	1.05	Intronic (Transcribed)	5,37,99,977	1.52 × 10^−9^		^24^
	rs8044769	*FTO*	C/T	1.11	Intronic (Transcribed)	5,38,39,135	6.85 × 10^−8^		^8^
	rs6499244	*NFAT5/WWP2*	A/T	1.06	3′UTR (Transcribed)	6,97,35,271	3.88 × 10^−11^		^24^
	rs34195470	*WWP2*	G/A	1.07	Intronic (Enhancer)	6,99,55,690	2.7 × 10^−11^		^22^
	rs864839	*JPH3*	T/G	1.08	Intronic (Repressed)	8,76,71,419	2.01 × 10^−8^		^21^
	rs1126464	*DPEP1*	G/C	1.04	Missense (Transcribed)	8,97,04,365	1.56 × 10^−10^		^24^
17	rs35087650	*SMG6*	TT/-	1.07	Intronic (Transcribed)	20,58,350	1.18 × 10^−9^		^24^
	rs8067763	*SOX9*	G/A	1.06	Intergenic (Repressed)	1,00,12,939	2.39 × 10^−9^		^24^
	rs2953013	*NF1*	C/A	1.05	Intronic (Transcribed)	2,94,96,343	3.07 × 10^−10^		^24^
	rs62063281	*MAPT*	G/A	1.10	Intronic (Repressed)	4,40,38,785	5.30 × 10^−12^		^24^
	rs547116051	*MAPT*	C/-	1.83	Intronic	4,40,57,887	1.50 × 10^−8^		^24^
	rs7222178	*NACA2*	A/T	1.09	Intergenic	5,96,52,282	1.70 × 10^−9^		^22^
			A/T	1.10			3.78 × 10^−11^		^24^
	rs2521349	*MAP2K6*	A/G	1.13	Intronic (Transcribed)	6,75,03,501	9.95 × 10^−10^		^21^
18	rs10502437	*TMEM241*	G/A	1.03	Intronic (Transcribed)	2,09,70,706	2.50 × 10^−8^		^24^
19	rs11880992	*DOT1L*	A/G	ns	Intronic (Transcribed)	21,76,403	3.20 × 10^−16^		^14^
	rs12982744	*DOT1L*	C/G	1.17	Intronic (Transcribed)	21,77,193	7.8 × 10^−9^		^10^
	rs1560707	*SLC44A2*	T/G	1.04	Intronic (Transcribed)	1,07,50,738	1.35 × 10^−13^		^24^
	chr19:18,898,330	*COMP*	G/C	16.70	Missense	1,88,98,330	4.00 × 10^−12^		^15^
	rs375575359	*ZNF345*	del/T	1.21	Intronic (Heterochromatin)	3,73,53,347	7.54 × 10^−9^		^21^
	rs75621460	*TGFB1*	A/G	1.16	Intergenic (Enhancer)	4,18,33,784	1.62 × 10^−15^	*TGFB1*	^24^
	rs4252548	*IL11*	T/C	1.30	Missense (Transcribed)	5,58,79,672	2.1 × 10^−11^		^22^
				1.32			1.96 × 10^−12^		^24^
20	rs143384	*GDF5*	A/G	1.10	5′UTR (Promoter)	3,40,25,756	1.40 × 10^−19^		^22^
				1.10			4.77 × 10^−23^		^24^
	rs143383	*GDF5*	T/C	1.79	5′UTR (Promoter)	3,40,25,983	1.8 × 10^−13^	*GDF5*	^2^
				1.17			6.2 × 10^−11^		^6^
	rs6094710	*NCOA3*	A/G	1.28	Intergenic (Enhancer)	4,60,95,649	2.0 × 10^−10^	*NCOA3*	^11^
21	rs6516886	*RWDD2B*	T/A	1.10	Intergenic (Transcribed)	3,03,93,664	5.84 × 10^−8^	*RWDD2B*	^21^
	rs2836618	*ERG*	A/G	1.09	Intergenic	4,00,48,295	3.20 × 10^−11^		^24^
22	rs117018441	*EP300*	T/G	5.89	Intergenic (Transcribed)	4,15,53,917	1.8 × 10^−25^		^22^
	rs532464664	*CHADL*	ins8/-	7.70	Missense (Promoter)	4,16,34,088	4.50 × 10^−18^		^15^
	rs528981060	*SCUBE1*	A/G	1.68	Intronic (Repressed)	4,36,62,241	2.0 × 10^−8^		^24^

Effect sizes of OA risk-conferring alleles are small, with the vast majority of odds ratios (ORs) being <1.5 and with no common large-effect loci of the magnitude seen, for example, at the human leucocyte antigen (HLA) in autoimmune arthritic diseases such as rheumatoid arthritis^26^. The largest OR so far reported for an OA locus is 16.70 to a variant within the cartilage oligomeric matrix protein gene *COMP*, but this variant is restricted to an extensive Icelandic pedigree and is absent from other European populations (Table I)^15^.

Overall, OA is an archetypal example of a common, polygenic disease in which disease occurs due to the inheritance of multiple risk alleles of modest individual impact. It also fits with the “liability threshold” model of polygenic diseases^27^. This model considers that many distinct genetic variants can increase susceptibility to a discontinuous disease or trait, such as whether an individual has OA or does not. If an individual reaches the threshold number of those variants and their concomitant effects are exerted, they will consequently develop the disease.

### OA risk alleles primarily reside in non-protein coding regions of the genome

**Fig. 1:**
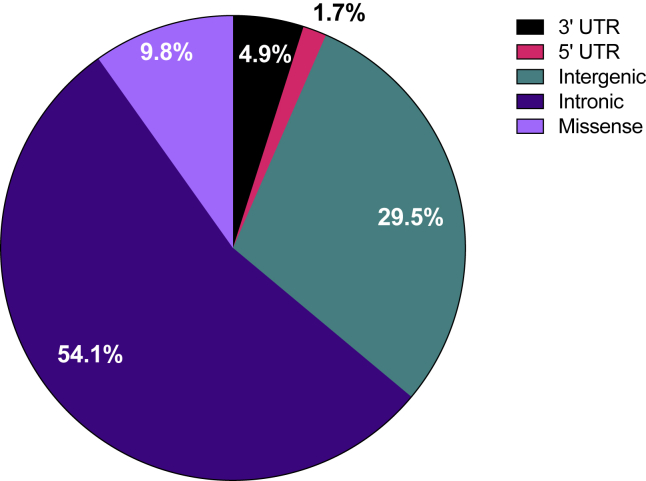
The genomic locations of the SNPs reported to be associated with OA. UTR, untranslated region.

There are two main mechanisms by which genetic variation can act on a phenotype. The first is through a direct change to a protein. This could, for example, be the result of a genetic variant changing the DNA code in the coding sequence of a gene, which introduces an amino acid substitution that alters protein function. This is the mechanism by which DNA variants act in many Mendelian diseases^28^ but is rare for common diseases. The second mechanism is through altering the regulation of gene expression, leading to an increase or decrease of the level of a gene's mRNA, and subsequently the levels of the encoded protein. This is the mechanism by which the vast majority of variants act in common diseases^29^. Genetic loci at which SNP genotypes are associated with such changes in gene expression levels are termed expression quantitative trait loci (eQTLs)^30^^,^^31^ and most of the genetic variants that have so far been associated with common diseases are located in non-coding regions of the genome^32^. In OA, less than 10% of the 124 SNPs so far associated with the disease are located in a protein coding sequence (Fig. 1).

### Functional follow-up of OA genetic signals – statistical fine mapping and *in silico* analysis

Across the field of common disease research, it has proven challenging biologically to interpret the results of GWAS and consequently the translation of genetic discoveries into effective therapies remains elusive^33^. Significant GWAS hits are reported in the form of lead, or “sentinel”, SNPs which mark a genomic locus associated with disease^34^. These loci can be large and complex regions of high linkage disequilibrium (LD), and this information alone provides little insight into causal variants, target genes, and the molecular mechanisms underpinning disease pathology.

Direct functional follow-up of the loci in relevant tissues and disease models is therefore essential, yet this process is laborious, expensive, and can require large numbers of patient samples. Furthermore, causal variants can exert their pathological effects in a spatiotemporal manner, and it is vital that appropriate investigative tools and models are applied. As a result, statistical methods of refining GWAS signals, along with the integration of genome and epigenome-wide public datasets are increasingly being applied post-GWAS to prioritise variants and genes for subsequent functional analyses.

Due to the small effect sizes conferred by SNP genotype in most polygenic diseases, simply selecting the SNP at each locus with the lowest *P*-value is unlikely to provide insight into the most probable causal variant^35^. Bayesian fine-mapping considers all SNPs at a single locus that reach genome-wide significance (*P* < 5 × 10^−8^) along with accompanying variants in LD^36^. Posterior probabilities (PP) of causality are applied to identify a credible set of variants, which can contain either single or multiple causal SNPs^36^^,^^37^. To date, fine-mapping approaches have been applied to several GWAS of OA^21^^,^^23^^,^^24^. This includes the largest OA GWAS to date, which mined the full UK Biobank dataset to investigate over 77,000 individuals with the disease^24^. At 6 of the 52 novel OA risk loci reported, the investigators were able to identify a single causal variant with >95% PP; SNPs rs34811474, rs13107325, rs547116051, rs75621460, rs4252548 and rs528981060 in Table I.

**Fig. 2:**
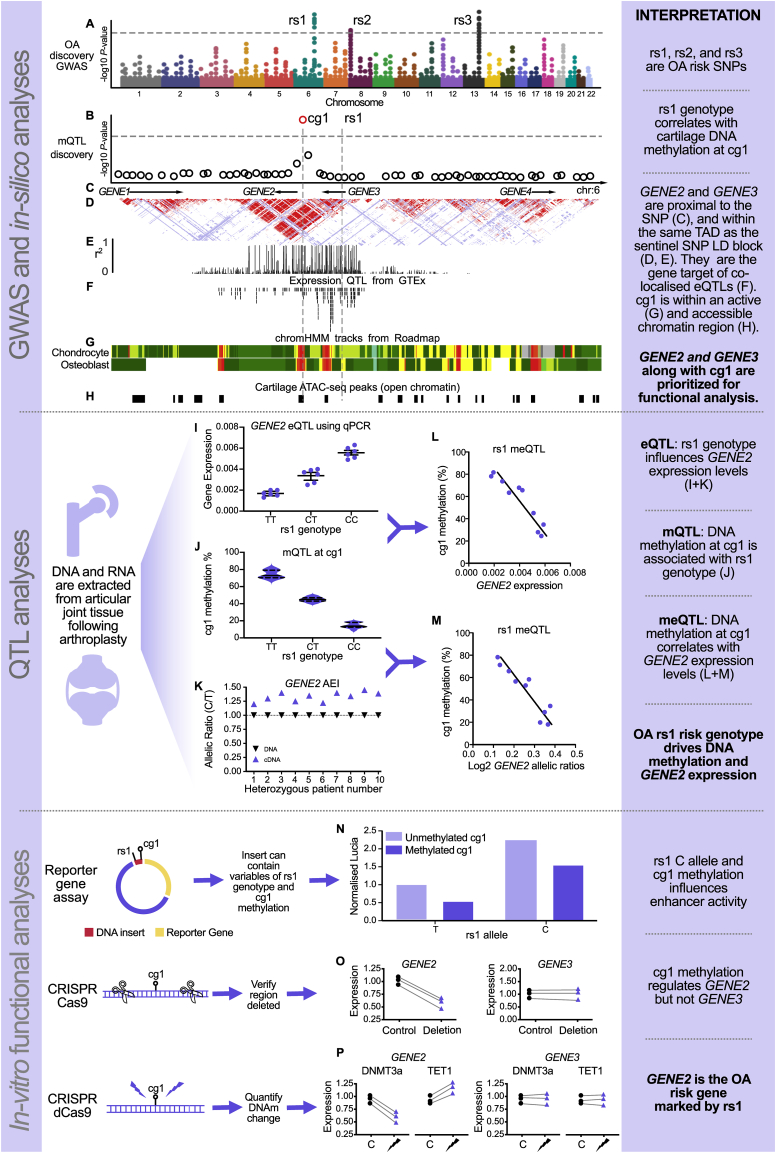
Example of how an OA GWAS risk SNP can lead to mechanistic insights and an associated OA risk gene. (**A**) Manhattan plot of an OA GWAS. Each dot represents a SNP. The -log10 *P*-value represents the significance of each SNP being preferentially carried by OA patients vs healthy controls. The dotted line represents the genome wide significance threshold. SNPs rs1, rs2 and rs3 are all OA significant risk SNPs. (**B**) Cartilage mQTL analysis for rs1. Each circle represents a CpG site available on the methylation array. The -log10 *P*-value represents the significance that methylation at a CpG is associated with rs1 genotype. The dotted line represents the statistical significance threshold after multiple testing correction. The red circle represents a CpG site (cg1) significantly associated with rs1 genotype (this is an mQTL). This mQTL analysis should be done for each SNP that is found to be associated with OA (rs2 and rs3). This analysis is usually limited to the CpGs within the physical proximity of each association SNP (e.g., 1 megabase (Mb) region). (**C**) Arrows representing the location and transcriptional direction of each gene within the region. rs1 is located within GENE3. (**D**) Linkage disequilibrium (LD) in CEU population from the HapMap project. Image correlates with the topologically associated domains (TADs), shown by each pyramid, within the region. Red represents regions in LD, with each pyramid encompassing a region that is inherited as a block. In this example both rs1 and cg1 are within the same TAD as are GENE2 and GENE3. These genes would be prioritized as candidate OA risk genes for further analysis. (**E**) LD link r^2^ values between rs1 and each SNP within the region. Each bar represents a SNP and the height corresponds to the r^2^ value between 0 and 1. All SNPs in high LD with rs1 are just as likely to be the functional SNP. (**F**) UCSC (http://genome.ucsc.edu/) track from GTEx showing all eQTLs operating within the region. Each line represents an eQTL in a tissue type for one of the four genes listed. The position of the bar on the *x*-axis represents which SNP this operates at. All SNPs with an eQTL are prioritized as functional. (**G**) UCSC track of ChIP-seq data in chondrocytes and osteoblasts. Green represents transcription sites, yellow enhancer sites and red transcription start sites. Enhancers and transcription start sites are prioritized as functional. (**H**) Genome wide ATAC-seq data for knee articular cartilage^40^ available on UCSC browser. (**I**) Association between genotype at rs1 and GENE2 expression levels using qPCR. Each dot represents cartilage tissue from a different patient. C allele is associated with increased expression. (**J**) Violin plot showing association between genotype at rs1 and methylation levels at cg1. The width of the blue violin represents the proportion of patients within that range, the bar the mean and the dotted line the quartile range. C allele at rs1 is associated with decreased methylation at cg1. (**K**) Allelic expression imbalance (AEI) of GENE2. rs1 C allele is preferentially transcribed compared to the T allele. (**L**) Association between GENE2 expression levels and cg1 methylation levels, highlighting a methylation-expression QTL (meQTL). Each dot represents cartilage tissue from a different patient. Increased GENE2 expression is associated with decreased cg1 methylation levels. (**M**) Association between GENE2 AEI and cg1 methylation, again highlighting an meQTL. An increased ratio of rs1 C/T allele transcripts is associated with decreased methylation at cg1. (N) In vitro Lucia reporter analysis with DNA insert containing rs1 and cg1. rs1 C and T alleles are compared to each other with cg1 methylated or unmethylated. C allele and decreased methylation act synergistically to increase enhancer activity. (O) In vitro deletion of cg1 region using CRISPR-Cas9 and subsequent gene expression analysis of GENE2 and GENE3. Each dot represents a biological *in vitro* replicate. GENE3 expression levels are unchanged whilst GENE2 expression levels are reduced. (P) In vitro methylation and demethylation of cg1 using CRISPR-dCas9 DNMT3a/TET1. On the *x*-axis, controls are listed by the abbreviation C and cases by the lightning bolt. Methylation changes do not influence GENE3 expression levels. Increased methylation of cg1 decreases GENE2 expression and conversely, decreased methylation increases expression levels. This concludes that methylation at cg1 is only driving GENE2 expression. GENE2 is deemed the target of the OA risk marked by SNP rs1.

Post-GWAS, the resolution of fine-mapping can be further enhanced by the integration of genomic and epigenomic functional data^36^. Again, this approach is being increasingly utilised in the OA field, aided in recent years by the generation and subsequent public availability of large datasets generated in human articular cartilage, chondrocytes, and mesenchymal stem cells (Fig. 2, sections A to H). Such datasets have proved invaluable to the OA and musculoskeletal research community and include gene and transcript expression data^38^^,^^39^, foetal and aged chromatin accessibility data (ATAC-seq)^40^^,^^41^, chromatin state data (ChIP-seq)^42^, long range chromatin interactions (Capture HiC)^43^, and DNA methylation (DNAm) data^44^, ^45^, ^46^, ^47^, ^48^. The inclusion of such data into post-GWAS analysis helps to assign a biological function to OA-associated variants. A prime example of the successful mining of such datasets post-GWAS was reported in a recent study of hand OA^25^. The integration of relevant datasets prioritised the Wnt ligand gene *WNT9A*, its regulatory elements, and causal variant rs1158850 at the locus^25^. Other public resources have been applied to the OA field to further support *in silico* analyses. This includes TRANSFAC^49^ to identify SNP-mediated changes to transcription factor binding motifs, and GTEx^30^ to colocalise eQTLs with associated SNPs^36^. However, as mentioned above, it is vital that such data integrations are correctly interpreted, as many public databases do not include data generated in articular joint tissues and, as such, the continuation of data generation in human musculoskeletal tissues is vital to advance the field.

### Functional follow-up of OA genetic signals – laboratory studies

Following statistical fine mapping and *in silico* analysis, focussed *in vitro* and *in vivo* studies of individual loci are essential to establish causal effects of allelic variation and to validate prioritised gene targets. In the OA field, a range of molecular genetic techniques have been applied for this purpose using patient joint tissues and relevant primary and immortalised cells. Arthroplasty samples of osteoarthritic joint tissues are invaluable to study the effect of genotype upon differential expression of genes (eQTLs) and to identify correlations between genotype and DNA methylation (mQTLs; see below). Due to interindividual variability in gene expression, it is often difficult to detect eQTLs through direct stratification of gene expression levels by SNP genotype. This observation is supported by the large numbers of patient samples required to identify such tissue-specific effects in the GTEx database^30^. A complementary approach, which is considerably more sensitive, is allelic expression imbalance (AEI) analysis, in which the relative ratio of mRNA transcripts produced from each allele of a SNP are quantified^12^^,^^18^^,^^50^, ^51^, ^52^, ^53^, ^54^, ^55^, ^56^, ^57^. This has proven useful in identifying or narrowing down the effector gene, or genes, at a gene-rich OA locus, such as at chr3p21.1, where significant AEI was identified in patient cartilage for five of the seven investigated genes, including the nuclear protein gene *GNL3* and the signal peptidase gene *SPCS1*^51^. A separate investigation of OA risk conferred by the T allele of rs4764133, identified a reduction in cartilage expression of the matrix Gla protein gene *MGP*, relative to the non-risk allele C, confirming this gene as the mediator of OA risk at this locus^18^. Of note, this effect was also observed in other joint tissues, whilst the opposite effect was observed in whole blood samples^55^, highlighting the tissue-specificity of the molecular mechanisms underlying disease risk and the biological pleiotropy exerted by risk SNPs.

Gene reporter assays have been widely applied to validate regions for regulatory activity *in vitro*, and can be adapted to investigate the impact of both SNP genotype and DNAm upon regulatory function^52^^,^^53^. Furthermore, such assays can be used to validate putative microRNA (miRNA) binding sites and miRNA gene targets. In 2019, a massively-parallel reporter assay (MPRA) was applied to investigate 35 known OA risk loci^58^. Klein and colleagues investigated all SNPs in LD at each locus, a total of 1,605, and observed significant differential allelic regulation at six variants^58^.

The advent of CRISPR-Cas9 and subsequent development of the Cas9 toolbox has revolutionised targeted editing of the genome and epigenome^59^. For functional analyses of OA risk loci, CRISPR has been used to delete both putative regulatory elements and functional SNPs in Tc28a2 chondrocytes, confirming the RUNX family transcription factor gene *RUNX2,* the transforming growth factor gene *TGFB1,* and the collagen galactosyltransferase gene *COLGALT2* as targets of OA genetic risk^52^^,^^60^^,^^61^. The development of a catalytically dead Cas9 (dCas9) fused to enzymes which either methylate (DNMT3a) or demethylate (TET1) CpGs has allowed precision editing of DNAm at targeted CpGs for the first time. This is increasingly being applied to cell models of OA, and has demonstrated causal relationships between CpG methylation and gene expression at loci where correlative relationships have previously been identified in patient samples^57^. A recent study focussing on the OA risk residing at chr21q21 used a dCas9-TET1 construct for targeted demethylation of a hypermethylated Methylation quantitative trait locus (mQTL) region within the promoter of the RWD domain-containing protein gene *RWDD2B.* A mean reduction in methylation of 21.5% within the cell population resulted in a 3.8-fold increase in gene expression, specific to *RWDD2B,* confirming the gene as the target of OA-associated genetic and epigenetic effects at the locus^57^.

Functional follow-up studies of GWAS results therefore enable the identification of target genes of OA associated SNPs. Table II lists some of the functional tools and approaches used in the analysis of risk loci whilst Fig. 2 provides a schematic of how one can progress from an association SNP (sections A to H) towards a functional variant (sections I to M) and target gene (sections N to P). The OA targets so far discovered via such approaches include *COLGALT2*, *RUNX2*, *PLEC* (encoding plectin), *MGP*, *ALDH1A2* (encoding aldehyde dehydrogenase), *TGFB1*, *GDF5* (encoding growth differentiation factor 5), and *RWDD2B* (Table I)^2^^,^^12^^,^^18^^,^^52^, ^53^, ^54^, ^55^, ^56^, ^57^^,^^60^^,^^61^. These genes encode proteins with diverse roles, including extracellular signaling molecule (*GDF5*, *TGFB1*), extracellular calcium regulator (*MGP*), intracellular enzyme (*ALDH1A2*), transcription factor (*RUNX2*) and cytoskeletal protein (*PLEC*), indicating that OA genetic risk is operating on a broad range of biological functions.

**Table II:** A list of functional tools and how they are used in OA genetic studies

Tool	Definition	Utility and interpretation of data
Linkage disequilibrium (LD)	The non-random association of two alleles within a population. LD ranges from 0 to 1. At perfect LD (*r*2 = 1) alleles are inherited together as a haplotype	SNPs in LD with the association SNP, and the genomic regions in which they reside, are prioritized for further investigation
ATAC-sequencing	Assesses chromatin accessibility at a genome-wide level in cells of interest	Open regions signify potential regulatory elements and are prioritized as functionally important
ChIP-sequencing	Categorizes DNA-protein binding regions into transcription start sites, enhancers or other active regulatory sequences	Regions labelled as active are prioritized as functionally important
Expression quantitative trait locus (eQTL)	SNP genotype correlates with expression of a gene. Can be measured by allelic expression imbalance (AEI) analysis or by stratifying expression of a gene by GWAS SNP genotype	If expression correlates with genotype, the gene may be the target of the functional effect mediated by the association signal
Methylation quantitative trait locus (mQTL)	SNP genotype correlates with methylation levels at a CpG	If methylation correlates with genotype, that CpG may be an intermediate of the functional effect mediated by the association signal
Methylation and expression quantitative trait locus (meQTL)	CpG methylation levels correlate with the expression of a gene	The GWAS SNP may act by differentially methylating DNA to regulate gene expression
Reporter analysis	in vitro experiment assessing reporter protein expression driven by a DNA insert cloned at a promoter or enhancer site	Can be used to confirm that a sequence is a regulator of gene expression, that the two alleles of a SNP have differential regulatory activity, and that these effects can be modulated by DNA methylation
CRISPR-cas9	Sequence-specific guide RNAs are used to delete or alter the sequence of a DNA region of interest in a cell line or primary cell to assess effect on gene expression	Can reveal a particular gene from amongst many as being the target of the association signal
CRISPR dCas9-DNMT3a/TET1	Sequence-specific guide RNAs are used to hyper- or hypo-methylate a DNA sequence to assess effect on gene expression	Can confirm the role of DNA methylation as an intermediate between an association signal and the expression of its target gene

**Fig. 3:**
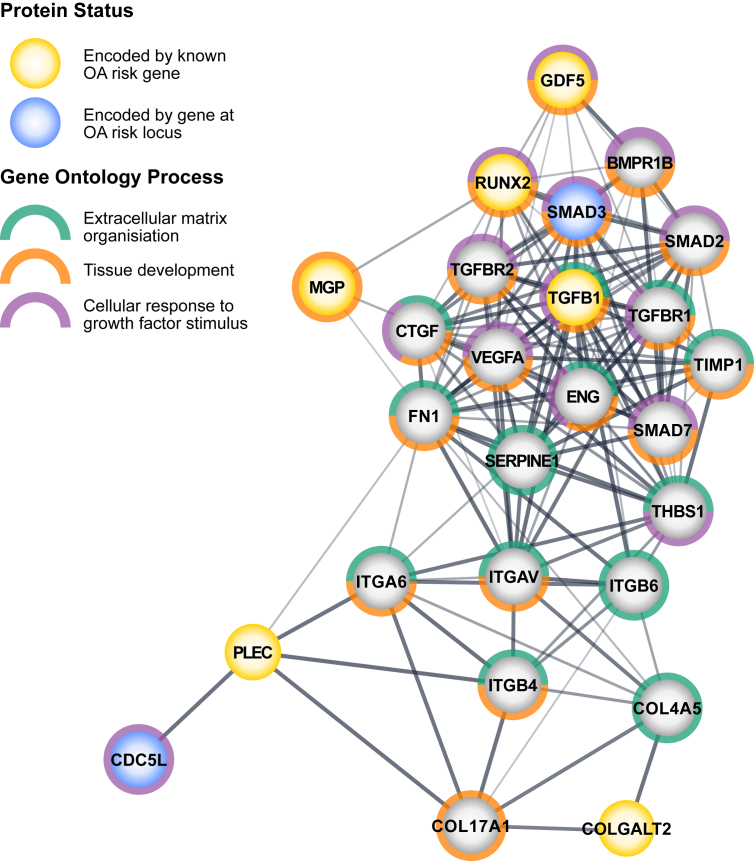
A STRING protein–protein interaction network of OA risk gene products. Yellow nodes indicate proteins encoded by OA risk genes *COLGALT2*, *RUNX2*, *PLEC*, *MGP*, *TGFB1* and *GDF5*. Blue nodes are proteins encoded by unconfirmed causal genes *SMAD3* and *CDC5L* at OA risk loci. Grey nodes are protein interactors. Nodes were functionally annotated with the top three Gene Ontology (GO) processes: extracellular matrix (ECM) organization, green (P = 5.23 × 10^−14^); tissue development, orange (P = 5.06 × 10^−13^); and cellular response to growth factor stimulus, purple (P = 1.63 × 10^−13^). Edge thickness (lines between proteins) indicates the confidence of interactions, based upon experimental evidence, co-expression, databases, and text-mining. STRING website; https://string-db.org/.

Of the above eight genes, six of the respective protein products fall within a network of protein interactions (Fig. 3). Interestingly, two additional proteins in this network, CDC5L (cell division cycle 5 like) and SMAD3 (SMAD family member 3), are also encoded by genes that reside at OA risk loci (Table I)^20^^,^^22^^,^^24^. This protein–protein interaction (PPI) network indicates that multiple variants on distinct chromosomes can lead to the dysregulation of genes that play vital roles in shared chondrocyte pathways. Furthermore, the network of OA risk gene products (PPI enrichment *P* = 1.0 × 10^−16^) is enriched for the Gene Ontology (GO; http://geneontology.org/) processes extracellular matrix organization, tissue development, and cellular response to growth factor stimulus (Fig. 3). Such PPI networks offer mechanistic insight into the liability threshold model of polygenic diseases mentioned earlier, by highlighting that when a pathway is impacted by multiple risk alleles, it is likely to be perturbed.

### Interaction between OA genetics and epigenetics

In recent years, the increase in the numbers of reported OA genetic risk variants, along with technological advances allowing in-depth epigenetic studies in cartilage, have uncovered an interplay between genetics and epigenetics in OA. This was recently the subject of an in-depth review^62^ and will only be discussed briefly here.

There are three main mechanisms of epigenetic regulation of gene expression: post-translational modification of histones, non-coding RNAs (ncRNAs, such as miRNAs), and DNAm, of which the latter is the most well-studied. Studies of the cartilage DNA methylome have led to the discovery of OA-associated methylation quantitative trait loci (mQTLs), at which there is a correlation between genotype at an OA risk SNP and *cis* DNAm^25^^,^^54^^,^^63^^,^^64^. DNAm is thought to act as a conduit through which the functional effect of SNP genotype is exerted upon expression of the target gene (Fig. 2). These analyses of the cartilage epigenome have allowed the epigenetic prioritisation of regulatory regions (harbouring the mQTLs), causal SNPs (located within the regions) and effector genes (regulated by the regions)^54^. We have recently repeated the mQTL analysis in our dataset of 87 hip and knee arthroplasty cartilage samples to include all OA GWAS SNPs reported to date, including both novel variants and those previously analysed (Table III). Of the 124 SNPs across the 95 independent loci reported in the literature (Table I), we were able to investigate 114, which were either directly genotyped on our array, or for which a suitable proxy was available. Genotype at all SNPs was investigated for correlations with CpG methylation in a 1 megabase (Mb) region flanking the association signal as previously described^54^. Twenty-eight SNPs were shown to mediate mQTLs across 23 different loci (some SNPs had shared effects upon DNAm at CpGs, Table III). This is consistent with our previous observations that 25% of OA risk loci are also mQTLs^54^^,^^63^^,^^64^.

**Table III:** Cartilage methylation quantitative trait loci (mQTLs) at OA association SNPs. The nearest protein coding gene to the mQTL is named. The physical location of the CpG (hg19 genome assembly) is reported. r2∗, linkage disequilibrium (LD) value between association SNP and the proxy SNP used for mQTL analysis; an r2 of 1.0 is perfect LD. r2∗∗, linkage disequilibrium value between the SNPs marking two mQTLs at a locus. rs6976/rs11177∗, SNPs are in perfect LD. FDR, *P*-values were adjusted to account for the tests performed using a false discovery rate (FDR) estimation based on Benjamini-Hochberg correction

Locus	GWAS SNP	Proxy SNP	r2∗	SNP chromosome	SNP position (hg19)	CpG ID	CpG position (hg19)	FDR	Slope	Gene	Reference	r2∗∗
1	rs11583641	rs10911472	1.00	1	18,39,06,245	cg18131582	18,39,12,305	2.50E-03	0.52	*COLGALT2*	^63^	
2	rs10916199	rs10799428	0.97	1	22,79,02,472	cg09796739	22,79,24,055	6.89E-07	0.38	*SNAP47*	^25^	
						cg11520395	22,79,24,115	6.83E-03	0.21			
3	rs62182810	rs2305417	0.94	2	20,43,87,482	cg10114877	20,44,27,199	4.66E-08	0.98	*RAPH1*	^63^	
4	rs6976/rs11177∗	–		3	5,27,28,804	cg18099408	5,25,52,593	3.85E-07	−0.79	*GNL3*	^64^	0.89
		–				cg13060642	5,25,56,643	2.86E-02	0.26			
		–				cg27294008	5,27,48,359	2.23E-02	0.23			
		–				cg18404041	5,28,24,283	2.51E-04	−0.21			
	rs678	–		3	5,28,20,981	cg18099408	5,25,52,593	4.43E-06	−0.76		Novel to this analysis	
		–				cg13060642	5,25,56,643	1.36E-02	0.28			
		–				cg27294008	5,27,48,359	2.95E-02	0.23			
		–				cg18404041	5,28,24,283	2.75E-06	−0.24			
5	rs11732213	rs798756	1.00	4	17,04,244	cg20987369	15,79,572	5.00E-03	0.39	*SLBP*	^63^	
				4		cg25007799	15,79,657	5.35E-03	0.73			
6	rs9277552	rs9277464	0.77	6	3,30,55,501	cg02197634	3,30,48,875	1.43E-02	0.96	*HLA-DPB1*	^63^	
						cg25491704	3,30,48,879	4.38E-03	0.86			
						cg13921245	3,30,53,791	6.21E-03	0.40			
						cg02375585	3,30,91,111	3.81E-02	−0.64			
7	rs10948155	rs529125	0.82	6	4,46,87,957	cg13979708	4,46,95,318	2.05E-04	−0.36	*SUPT3H/RUNX2*	Novel to this analysis	0.60
						cg20913747	4,46,95,427	3.56E-07	−0.66			
	rs10948172	–		6	4,47,77,691	cg13979708	4,46,95,318	2.05E-04	−0.36		^64^	
		–				cg20913747	4,46,95,427	3.56E-07	−0.66			
8	rs1997995	rs9296459	0.94	6	4,53,74,183	cg25494480	4,53,87,917	4.34E-02	0.32	*SUPT3H/RUNX2*	Novel to this analysis	
9	rs60890741	rs12542856	0.96	8	13,07,68,504	cg18170545	13,10,80,137	1.59E-02	−0.35	*ASAP1*	^63^	
10	rs11780978	–		8	14,50,34,852	cg19405177	14,50,01,428	2.48E-17	0.77	*PLEC*	^54^	
		–				cg20784950	14,50,02,522	1.31E-05	0.47			
		–				cg01870834	14,50,02,835	1.09E-05	0.38			
		–				cg24891660†	14,50,03,653	3.81E-02	0.42			
		–				cg07427475	14,50,08,110	6.18E-18	−1.24			
		–				cg02331830	14,50,08,288	4.48E-08	−0.99			
		–				cg04255391	14,50,08,397	8.46E-16	−0.84			
		–				cg14598846	14,50,08,909	7.25E-22	−1.24			
		–				cg23299254	14,50,08,957	2.81E-18	−1.24			
		–				cg10299941	14,50,09,137	3.44E-02	−0.33			
		–				cg21511203†	14,50,15,037	2.93E-02	0.25			
		–				cg07212837†	14,50,68,773	4.34E-02	−0.11			
11	rs2070852	–		11	4,67,44,925	cg03339077	4,71,65,057	7.53E-03	0.38	*F2*	^25^	
12	rs10896015	rs12270054	1.00	11	6,53,23,725	cg21890820	6,53,08,645	4.45E-02	0.38	*LTBP3*	Novel to this analysis	
13	rs4764133	rs11614333	0.98	12	1,50,64,363	cg20917083	1,51,14,233	1.76E-02	0.27	*MGP*	^63^	
14	rs317630	rs490872	1.00	12	6,96,37,847	cg22375663	6,97,25,435	1.31E-11	1.06	*CPSF6*	^63^	
15	rs1060105	–		12	12,38,06,219	cg21745287	12,34,64,511	2.23E-02	0.39	*SBNO1*	Novel to this analysis	0.12
		–				cg10169515	12,37,07,536	6.89E-07	0.77			
	rs56116847	rs28466887	0.96	12	12,38,35,233	cg10169515	12,37,07,536	4.36E-02	−0.46		Novel to this analysis	
16	rs3204689	rs3204689		15	5,82,46,802	cg12031962	5,83,53,849	1.12E-07	−0.62	*ALDH1A2*	^64^	
17	rs35206230	rs1378940	1.00	15	7,50,97,780	cg20040747	7,47,15,105	1.97E-03	−0.29	*CSK*	^63^	
						cg10253484	7,51,65,896	1.10E-02	0.36			
18	rs6499244	rs1364063	0.90	16	6,97,35,271	cg26736200	6,99,51,706	1.06E-02	−0.48	*WWP2*	^63^	0.26
						cg26661922	6,99,51,820	6.16E-03	−0.45			
	rs34195470	rs904809	0.79	16	6,99,55,690	cg26736200	6,99,51,706	1.20E-26	−1.02		Novel to this analysis	
						cg26661922	6,99,51,820	4.85E-22	−0.90			
19	rs2953013	–		17	2,94,96,343	cg13263104	2,96,71,293	4.86E-02	−0.33	*NF1*	^63^	
20	rs62063281	rs16940665	1.00	17	4,40,38,785	cg17117718	4,36,63,208	1.20E-26	1.65	*MAPT*	^63^	
						cg10826688	4,37,14,992	1.07E-03	−0.35			
						cg15295732	4,39,42,128	3.89E-04	−0.39			
						cg11117266	4,39,71,461	5.30E-03	−0.46			
						cg16520312	4,39,71,471	2.26E-03	−0.31			
						cg18878992	4,39,74,344	1.95E-14	1.40			
						cg18228076	4,39,83,362	7.91E-11	−1.10			
						cg07368127	4,42,30,994	4.34E-02	0.44			
						cg15633388	4,42,66,530	1.16E-18	−1.52			
						cg23616531	4,42,69,258	5.20E-05	−0.41			
21	rs10502437	–		18	2,09,70,706	cg15535896	2,10,79,381	1.68E-02	1.20	*TMEM241*	Novel to this analysis	
22	rs143383	rs6087704	0.91	20	3,40,25,756	cg14752227	3,40,00,481	8.54E-03	0.78	*GDF5*		
23	rs6516886	rs2832155	1.00	21	3,03,93,664	cg18001,427	3,03,91,784	3.81E-02	−0.53	*RWDD2B*	^54^	
						cg20220242	3,03,92,188	1.05E-05	−0.68			
						cg24751378	3,03,96,349	3.09E-04	−0.23			
						cg16140273	3,04,55,616	1.86E-02	0.34			

Several OA risk loci have been identified which colocalise with genes encoding histone-modifying proteins including the transcriptional regulator *SUPT3H,* the nuclear receptor coactivator *NCOA3* and the histone methyltransferase *DOT1L*^8^, ^9^, ^10^, ^11^^,^^14^. *DOT1L* expression is essential for cartilage homeostasis, and the identification of this region as a risk locus strongly supports the requirement for tight regulation of chromatin state to maintain cartilage integrity^62^. Similarly, OA risk SNPs have been identified in the region of cartilage-specific ncRNAs that are known to be vital for homeostasis of the articular joint surface and are dysregulated in OA, including the miRNAs miR-140 and miR-455^23,25^.

Our knowledge of the interplay between genetics and epigenetics is rapidly developing as novel technologies emerge that allow interrogation of the epigenome. Future analyses require in-depth studies utilising large sample sizes to further enhance our understanding of the role of epigenetics as a contributor to OA development.

### Evolutionary origins and developmental effects of OA genetic risk

One of the most fascinating investigations of OA genetics in recent years has been into the role of natural selection as humans adapted to bipedalism and its relationship to OA risk. As noted above, the vast majority of OA SNPs reside in non-coding regions of the genome and are presumed to regulate gene expression. In a series of publications, Capellini and colleagues have shown that a large proportion of these SNPs are enriched in chondrocyte regulatory elements that are active during joint formation in the embryo, that these elements have been subjected to selection to facilitate bipedalism, and that their DNA sequences have then been constrained to further changes to preserve the derived joint shape^41^^,^^65^, ^66^, ^67^. It would appear, therefore, that a proportion of OA risk alleles are subtly changing these regulatory elements (for example, by quantitatively altering the binding of gene regulatory proteins), that this is tolerated due to only modest effects on joint morphology but that these changes become pathological and detrimental as we age. Since these negative effects only impact the elderly, there is no selective pressure acting against the alleles. It is proposed that these OA risk alleles have achieved appreciable frequency in the population by drift or by antagonistic pleiotropy^41^. The latter is when an allele has a positive phenotypic effect in the young but a negative phenotypic effect in the elderly and is a process thought to contribute to the occurrence of many common age-associated diseases^68^. These deleterious effects of allelic drift and of pleiotropic alleles will become increasingly important to public health as populations age and the prevalence of OA continues to increase.

The concept that OA has etiological routes in joint development is not a new one^69^, ^70^, ^71^, ^72^ and many studies have considered the role of OA SNPs as modulators of joint shape^73^, ^74^, ^75^, ^76^, ^77^. However, the data from the Capellini team provides evidence that a proportion of OA genetic risk is instigated at the start of life. This has profound implications in that joint development may be as important, if not more so, than joint maintenance when considering fundamental causes of the disease. There are also consequences for the functional and genomic studies of OA GWAS signals. At present, when primary tissues are investigated these tend to be donated by OA patients who have undergone arthroplasty. In future, developmental tissues should also be analyzed. This will enable further understanding of the interaction between our DNA and its own self-regulatory mechanisms, how this relationship adapts throughout the life-course, and when and how functional effects are exerted, leading to disease. As an example, one could examine foetal tissue to assess if OA associated eQTLs identified in OA patients are operating during development. If so, this would imply that the gene regulatory elements that are the targets of OA genetic risk are also functionally active during development and that they then go on to predispose to OA as we age.

### Clinical utility of OA genetics

Although the biological complexity of OA is daunting, the progress being made in our molecular genetic understanding of the disease has the potential to inform and accelerate development of prognostic markers and tailored OA therapeutics^78^. Indeed, several disease modifying osteoarthritis drugs (DMOADs) that are currently in clinical trials, including intra-articular TGF-β and FGF-18 growth factor therapies and Wnt inhibitors^79^, are targeting proteins whose genes have been highlighted by GWAS^21^^,^^24^.

The link between OA genetics and epigenetics opens up the latter as a therapeutic option. As noted earlier, several OA risk loci colocalise with genes encoding histone-modifying proteins^8^^,^^9^^,^^10^^,^^11^^,^^14^ and histone deacetylase (HDAC) inhibitors do show efficacy as inhibitors of the expression of catabolic molecules, such as matrix metalloproteinases (MMPs) and IL-1, in OA chondrocyte and mouse OA models^80^. The ability to modulate DNAm using CRISPR-Cas9 tools is also promising. In the functional study cited above, a dCas9-TET1 construct was used for demethylation of a hypermethylated mQTL within the promoter of *RWDD2B*^57^. The resulting increase in expression of *RWDD2B* reversed the impact of the OA genetic risk at this locus. Although undertaken in an immortalized cell line, this study highlights the possibility of using epigenome editing to counter the gene expression effects of a risk locus.

The use of DMOADs and epigenetic interventions will need to consider how best to deliver a therapeutic into the joint tissue and ensure its permanency, and which patients to target for which therapies^79^. This latter issue may be aided by the application of OA polygenic risk scores (PRS). As ever more GWAS loci are reported for a given disease, there is an enhanced ability to stratify an individual's probability of developing disease based upon frequency and effect size of their inherited variants, generating a PRS^81^. The development of PRS has the potential to accurately screen for disease risk among populations and can inform interventions^82^^,^^83^.

There are therefore reasonable grounds to be optimistic that the data emerging from OA genetic studies, and from the genomic analyses that complement these, will be applied to patient treatment^84^. Based on the likely developmental origin of some OA risk, translation of genetic discoveries will need to consider the time in an individual's life at which it is most optimal to start a treatment.

## Concluding remarks

Many OA genetics reviews have been written over the years and each new one provides further insight into this common, complex disease. This reflects the speed at which the field is advancing, with new discoveries being reported on a regular basis. The bedrock of this is the highly successful application of GWAS to large cohorts, which have so far yielded over 100 OA GWAS SNPs (Table I). From these studies, it is apparent that OA genetic risk is operating on a range of biological mechanisms encompassing articular joint formation, homeostasis and maintenance (Fig. 3), with impacts on the expression of synovial joint tissue genes being common.

Although impressive, the current proportion of the heritability accounted for by known OA risk loci is just over 20%^24^, meaning that there are still a large number of loci to be discovered. Efforts are underway to fill the gap and at an impressive scale, with large cohorts from across the globe being investigated (https://www.genetics-osteoarthritis.com/home/index.html). What is particularly exciting about these studies is that ethnic groups who have been underrepresented in OA GWAS are now starting to be included, which should enable a clearer picture to be generated of the genetic architecture of OA at a more global population level. Ongoing GWAS should continue to be complemented by follow-up genomic data analyses. This should not be restricted to cartilage nor to a particular age group: as touched on above, the molecular genetics of OA should be considered as a risk running throughout the life-course and not restricted to older individuals.

Although for many loci it is possible to highlight a causal SNP and then prioritize a target gene by statistical fine mapping and *in silico* analysis, this is not always the case. The application of laboratory-based functional tools is essential to validate a target and to elucidate one when fine-mapping draws a blank. Going forward, the use of cells with enhanced chondrogenic potential^85^ combined with three dimensional (3D) models of cartilage, and of cartilage with bone^86^, will provide more robust and realistic cell and organ models for the functional analyses of OA SNPs and their target genes. These models will also enable the inclusion of mechanical load as an experimental parameter, further aligning them with the *in vivo* reality of an articulating joint^87^. A complementary approach to such *in vitro* functional studies is the investigation of risk alleles in mice. This has so far proven particularly insightful for the OA risk that maps to the *GDF5* locus^41^ and similar reports are appearing in the literature^88^. A recent database of genes associated with OA in animal models can complement these investigations^89^. The degree to which the functional characterization of OA GWAS signals will benefit from animal models is open to debate, but clearly there are grounds for optimism^88^^,^^90^.

Finally, although no OA genetic study has yet led to a diagnostic tool or treatment, some of the OA-associated genes encode proteins that are active in pathways which have therapeutics available (Fig. 3), several of which are in clinical trials^21^^,^^24^. Genetic discoveries do therefore have translatable potential and as more GWAS loci are reported, the current gap between discovery and utility will narrow.

## Author contributions

All authors were involved in drafting the article and revising it critically for intellectual content, and all authors approved the final version to be submitted.

## Conflict of interest

The authors declare that they have no conflicts of interest related to the content of this manuscript.
